# Neural Stem Cells and Methods for Their Generation From Induced Pluripotent Stem Cells *in vitro*

**DOI:** 10.3389/fcell.2020.00815

**Published:** 2020-10-08

**Authors:** Adelya A. Galiakberova, Erdem B. Dashinimaev

**Affiliations:** ^1^Faculty of Biology, Lomonosov Moscow State University, Moscow, Russia; ^2^Center for Precision Genome Editing and Genetic Technologies for Biomedicine, Pirogov Russian National Research Medical University, Moscow, Russia; ^3^Koltzov Institute of Developmental Biology of the Russian Academy of Sciences, Moscow, Russia

**Keywords:** pluripotent stem cells, ESC, iPSC, neural stem cells, NSC, neurogenesis, regenerative medicine

## Abstract

Neural stem cells (NSCs) provide promising approaches for investigating embryonic neurogenesis, modeling of the pathogenesis of diseases of the central nervous system, and for designing drug-screening systems. Such cells also have an application in regenerative medicine. The most convenient and acceptable source of NSCs is pluripotent stem cells (embryonic stem cells or induced pluripotent stem cells). However, there are many different protocols for the induction and differentiation of NSCs, and these result in a wide range of neural cell types. This review is intended to summarize the knowledge accumulated, to date, by workers in this field. It should be particularly useful for researchers who are beginning investigations in this area of cell biology.

## Introduction

The *in vitro* generation of neural stem cells (NSCs) is a very attractive direction in terms of studying the processes of neural induction and the differentiation of progenitors into different types of neurons. Since the study of human embryonic neurogenesis is difficult for ethical reasons, the need to develop various models of *in vitro* neurogenesis is increasing. In addition, artificially obtained NSCs provide the opportunity to model various diseases of the central nervous system (CNS) and to study their pathogenesis and the methods for their treatment. Neural stem cell cultures can also be used as test systems for the screening of suitable drug candidates and for studying their effects on human nervous system cells. Finally, NSCs have a promising potential application in regenerative medicine by providing the opportunity for cell therapy of neurodegenerative diseases.

The most suitable source of NSCs *in vitro* is from cultures of pluripotent stem cells (PSCs). pluripotent stem cells are characterized by their long-term ability to self-renew and their potential for differentiation into any type of cell characteristic of the three germ layers. Since these cells have unlimited proliferative potential, it is possible to maintain them in culture under certain conditions for many years. There are two main types of PSCs: embryonic stem cells (ESCs) and induced pluripotent stem cells (iPSCs). These two types of PSC are largely similar to each other: gene expression profiles, morphology, telomerase activity, etc. (Okita et al., 2007). One source of ESCs is the cells of the inner cell mass of the embryo at the blastocyst stage (Evans and Kaufman, 1981). However, since obtaining ESCs in this way is therefore associated with manipulations of embryos, using such human ESCs (hESCs) is difficult for ethical reasons. Induced pluripotent stem cells can be generated by genetic reprogramming of somatic cells, and can thus provide a “best alternative” to ESCs. The first mouse iPSCs were obtained from fibroblasts in the Yamanaka laboratory, using retroviral transfection of the pluripotency genes (*Oct3/4, Sox2, c-Myc*, and *Klf4*) (Takahashi and Yamanaka, 2006). Later human iPSCs (hiPSCs) were obtained using the same set of pluripotency-associated genes (Takahashi et al., 2007). Subsequently, the technologies for obtaining hiPSCs have been progressively optimized, making it possible to obtain them from other types of cells: human keratinocytes, human peripheral blood cells, adipocytes, hepatocytes, stomach cells, etc. In addition, various methods of inducing pluripotent cells without using viral vectors have been proposed, meaning that such hiPSCs can be more suitable for cell therapy (Kaji et al., 2009; Aronovich et al., 2011).

Today, there is a wide variety of different methods of neural induction of PSCs. The protocols differ not only in the duration and cultivation conditions but also in the characteristics of the generated NSCs. In this review, we have aimed to highlight the most important studies in this area, and to describe the variety of neural induction protocols with their advantages, and disadvantages.

## Neural Stem Cells

In embryogenesis, development of the neural system starts with neural tube formation. Primitive dorsal ectoderm differentiates into to neural ectoderm, which then transforms into the neural tube during the process of “neurulation.” The lumen of the neural tube is composed of a neuroepithelium-forming ventricular zone (VZ; Gilbert, 2010). Dorso-ventral and antero-posterior patterning are very important events in neural tube maturation and specification. The gradients of various morphogens determine neuroaxis formation. The Wnt family, retinoic acid (RA) and fibroblast growth factor (FGF) act as “posteriorizing signals” (Cox and Hemmati-Brivanlou, 1995; Blumberg et al., 1997; Kiecker and Niehrs, 2001), while their antagonists Cerberus and Dickkopf mark the anterior region, which subsequently forms the forebrain, midbrain, and hindbrain (Piccolo et al., 1999; del Barco Barrantes et al., 2003). The main factors initiating dorso-vetral axis formation are the ventral marker Sonic Hedgehog (SHH), which is produced by axial mesoderm, and the dorsalizing factors – the transforming growth factor b (TGF-β) family [including Bone morphogenetic proteins (BMPs) and activin] that are produced by the adjacent non-neural ectoderm, and the Wnts family (Liem et al., 1995, 1997; Backman et al., 2005; Bonner et al., 2008; Dessaud et al., 2008).

Neural stem cells is the term commonly applied to multipotent undifferentiated cells, capable of generating all types of neurons, oligodendrocytes, and astroglia. These cells are capable of self-renewing (Kempermann et al., 2015). Neural stem cells play a major role both in embryonic development and in adult neurogenesis. In accordance with the hypothesis put forward in the review by Alvarez-Buylla et al. (2001), there are several types of cells, present during embryonic neurogenesis that can be called NSCs. These include cells of the neuroepithelium – epithelial cells of the VZ of the neural tube, possessing apical-basal polarity. Self-renewal in these cells occurs through symmetrical divisions (Williams and Price, 1995), whereas asymmetric cell divisions generate one daughter stem cell and a second, more differentiated NSC, or a neuron (Noctor et al., 2008; Shitamukai et al., 2011). Additionally, there are the radial glial cells (RGCs). These arise from neuroepithelial cells (Malatesta et al., 2000). Radial glial cells contain glycogen granules and have a morphology similar to astroglial cells, but also possess properties of the neuroepithelium. Thus they have an apical-basal polarity and exhibit expression of neuroepithelial proteins such as Nestin and Vimentin as well as glial markers including glial fibrillary acidic protein (GFAP) and astrocyte-specific glutamate transporter (GLAST; Bignami and Dahl, 1974; Gadisseux and Evrard, 1985; Shibata et al., 1997; Götz et al., 1998; Hartfuss et al., 2001). This type of NSC can directly or indirectly generate the majority of neurons in the brain (Malatesta et al., 2003; Noctor et al., 2004). Like neuroepithelial cells, RGCs are capable of both asymmetric and symmetrical divisions. Asymmetric divisions give rise to a “daughter” RGC and a more differentiated basal (intermediate) progenitor cell or a differentiated neuron (Noctor et al., 2004). Radial glial cells are also capable of generating glial precursors, but this occurs mainly in the late stages of embryonic neurogenesis after the main wave of neuron production (Malatesta et al., 2000, 2003). The third type of NSC is the basal (intermediate) progenitor cell (IPC). Originated from RGCs, this type represents a multipolar non-stem cell that undergoes one or two symmetrical divisions, and then differentiates into a neuron. IPCs express the *Tbr2*, *Cux1*, and *Cux2* genes, but not *Pax6*, and are committed to generating only neurons (Hirabayashi et al., 2004; Haubensak et al., 2004; Kowalczyk et al., 2009).

In the adult, brain neurogenesis occurs in the subventricular zone (SVZ) of the lateral ventricle and the subgranular zone (SGZ) of the hippocampal dentate gyrus. Neural stem cells, represented by radial glia-like (RG-like) cells, are located in these zones. Similar to the RGCs of the developing embryonic brain, NSCs of the SVZ and SGZ express Pax6, GFAP, GLAST, Nestin and Vimentin, as well as expressing Sox2 (Ferri et al., 2004; Urbán and Guillemot, 2014). However, they differ from RGCs in their lower proliferation rate (Simons and Clevers, 2011). Neural stem cells of the SVZ (type B cells) can divide, giving rise to neuronal precursors – transient amplifying cells (type C cells), which in turn differentiate into neuroblasts (Type A cells) (Doetsch et al., 1999). Type C cells – GFAP- and Vimentin-negative cells – are characterized by the gene expression of such transcriptional factors as *Mash1*, *Pax6*, and *Dlx2* (Doetsch et al., 1997). Type A cells are the closest precursors to neurons. This cell type remains Nestin-positive and GFAP- and Vimentin-negative but differs from the precursor in its expression of polysialylated neural cell adhesion molecule (PSA-NCAM), doublecortin (DCX), and TuJ1 (β-tubulin) (Doetsch et al., 1997; Francis et al., 1999). Neural stem cells from the SGZ are called Type I cells and can generate proliferating IPCs, called Type 2 cells, similar to the type B cells of the SVZ. Intermediate progenitor cells give rise to neuroblasts (Type 3 cells) (Seri et al., 2004; Sugiyama et al., 2013).

The mechanism of mammalian, and in particular human, neurogenesis remains unclear. There are still many questions about the sequences of neurogenic differentiation and progenitor cell lines, and about the origins and differences of the types of NSC. However, several factors and signaling pathways involved in neurogenesis *are* known today.

The Wnt-β-catenin pathway (canonical Wnt pathway) takes part in the regulation of cell cycle, proliferation, and differentiation (Reya et al., 2003; Cajánek et al., 2009; Davidson et al., 2012; Hadjihannas et al., 2012). The Wnt-β-catenin pathway is especially significant in neurogenesis. It has been shown that canonical Wnt-signaling regulates the progression of differentiation of IPCs into neurons *in vitro* and *in vivo* (Hirabayashi et al., 2004; Munji et al., 2011). At the same time, the Wnt-β-catenin pathway promotes self-renewal of RGCs, thus maintaining the radial glial population (Wrobel et al., 2007).

The SHH signaling protein plays a role in embryonic patterning of the CNS and regulates the cell cycle of neural stem and progenitor cells (Dahmane and Ruiz i Altaba, 1999; Wallace, 1999). SHH-signaling is also involved in the proliferation and maintenance of the NSCs of the adult SGZ and SVZ (Machold et al., 2003). It has been shown that exogenous SHH *in vitro*, or its overexpression in the SGZ *in vivo*, promotes proliferation of progenitor cells (Lai et al., 2003). Repression of SHH-signaling in SGZ Type 2 cells causes a decrease in their divisions and premature differentiation (Balordi and Fishell, 2007).

Bone morphogenetic proteins are a group of growth factors, a subgroup of the (TGF-β superfamily of signaling ligands. There are a lot of BMPs, acting via binding to three types of BMP receptors (BMPR Ia, Ib, and II) (Massague, 1998). Canonical BMP-signaling involves the Smad proteins (Heldin et al., 1997; Zhang et al., 1997). Noggin is the main inhibitor of BMPs and the BMP-signaling regulator in neurogenesis (Smith and Harland, 1992; Pera et al., 2004; Bonaguidi et al., 2008). BMP-signaling is involved in the regulation of neurodevelopmental processes (progenitor proliferation, differentiation, and apoptosis) (Hegarty et al., 2013). Repression of BMP-Smad-signaling is necessary for primary neural induction in naïve ectoderm during embryogenesis (Pera et al., 2003; Liu and Niswander, 2005). After the closure of the neural tube BMP4- or BMP7-activated BMP-Smad-signaling is required for neural crest induction (Liem et al., 1995). BMP-Smad1/5/8-signaling plays a role in neuronal differentiation, maturation, and specification in the embryonic CNS (Hegarty et al., 2013). Bone morphogenetic proteins are also involved in neural processes in the adult brain, but their effects may be different under various conditions. BMP-Smad-signaling supports the dormant condition of the NSCs of the dentate gyrus by reversibly reducing proliferation, thus saving their undifferentiated state (Mira et al., 2010).

Another member of the TGF-β superfamily – Nodal – seems to be involved in controlling neural fate specification in embryonic development. The TGF-β/Activin/Nodal pathway is required for the self-renewal of ESCs and maintaining their pluripotency (James et al., 2005). Due to the association of this pathway with ESC pluripotency, it potentially inhibits their neuroectodermal differentiation (Vallier et al., 2004). The Nodal antagonists Lefty1 and Cerberus-1 are required for anterior neural patterning (Perea-Gomez et al., 2002).

The FGFs are a family of signaling growth factors – mitogens that, in particular, participate in the embryonic development of the neural system (Vaccarino et al., 1999). Fibroblast growth factors are involved in NSC proliferation and neurogenesis. In mice, FGF-2 induces the proliferation of neuroepithelial cells and neural precursors that can be isolated from the embryonic spinal cord, telencephalon and mesencephalon (Murphy et al., 1990; Raballo et al., 2000). Furthermore, FGF-2 is able to stimulate the survival of NSCs in the presence of insulin−like growth factor−1 (IGF-1; Drago et al., 1991). Fibroblast growth factor-2 is also involved in the neural induction of PSCs *in vitro* (Kunath et al., 2007; Stavridis et al., 2007). Several studies on isolated embryonic and adult mouse NSCs have revealed that FGF-2 and epidermal growth factor (EGF) are involved in their proliferation (Reynolds et al., 1992; Vescovi et al., 1993; Gritti et al., 1996; Reynolds and Weiss, 1996). It turns out that sensitivity to FGF appears by the neural plate stage, with EGF being present at a later stage. Both factors can independently cause the proliferation of NSCs found in the early stages of neurogenesis (Tropepe et al., 1999).

There are several marker proteins and genes, the presence or expression of which in the cell is a necessary factor in its assignment to a pool of NSCs.

RNA-binding protein, Musashi homolog 1 (Musashi-1). This protein is involved in Notch-signaling regulation, promotes self-renewal of NSCs and keeps them from differentiating (Wakamatsu et al., 1999; Imai et al., 2001). A high level of expression of Musashi-1 protein has been detected in neural precursor cells, including NSCs in the embryonic CNS, so this makes Musashi-1 a marker for neural stem/progenitor cells (Kaneko et al., 2000).

Pax6 is a neuroectodermal marker. It begins to be expressed by neuroepithelial cells at the stage of the neural plate, but later, its expression remains mostly in the dorsal forebrain (Schwarz et al., 1999). Pax6 expression has also been detected in RGCs of the developing brain as well as in early neural progenitors and RG-like cells of the SGZ and SVZ of the adult brain (Heins et al., 2002; Maekawa et al., 2005).

Sox1 transcription factor is involved in the early stages of neurogenesis. Sox1 is considered the earliest specific marker of the neuroectodermal lineage. *Sox1* expression is also detected in adult neural progenitor cells and several types of neurons (Kan et al., 2007; Venere et al., 2012).

Sox2 is critical for maintaining the self-renewal and pluripotency of ESCs. It is also necessary for the proliferative ability of NSCs and the inhibition of neuronal differentiation of CNS progenitors (Graham et al., 2003). *Sox2* expresses in proliferating neural progenitors of the embryonic brain and in adult neurogenic zones (Ellis et al., 2004).

Nestin is a type VI intermediate filament protein, mostly expressed in the neural progenitor and stem cells of developing and adult brains (Mignone et al., 2004).

Neural cell adhesion molecule (NCAM) mediates cell-cell interactions and cell adhesion (Seki and Arai, 1993). Polysialylated neural cell adhesion molecule is one of the glycoforms of NCAM, modified by the addition of polysialic acid (PSA). Polysialic acid reduces the adhesive efficacy of NCAM, thereby participating in negative regulation of cell interactions (Seki and Arai, 1991). It has been shown that developing neurons express PSA-NCAM during their migration, neurite outgrowth and synaptogenesis. Its expression was also found in various zones of neural proliferation (including in the SVZ and SGZ of adult brain). Polysialylated neural cell adhesion molecule is a marker for neuroblasts – the closest to neurons neural progenitors during brain development (Amoureux et al., 2000; Vutskits et al., 2006; Gascon et al., 2010). NCAM, by contrast, is expressed in transient amplified cells and RGCs (Gascon et al., 2010).

Forkhead box protein G1 (FoxG1) is also known as Bf1 (forebrain-restricted transcription factor). It is a transcription factor that participates in forebrain development, regulates neuroepithelium proliferation and differentiation into neural progenitors (Xuan et al., 1995; Dou et al., 1999). Expression of FoxG1starts in the rostral part of the neural tube and continues in the telencephalic neuroepithelium and telencephalic neural progenitors (Dou et al., 1999).

The Emx1 and Emx2 – homeobox – proteins, are transcription factors, expressed in forebrain progenitors. *Emx2* expression is a marker for both the dorsal and ventral telencephalon and diencephalon, whereas *Emx1* expression is restricted to the dorsal telencephalon (Boncinelli et al., 1995). Expression of both *Emx* genes is also distributed among telencephalon structures in the postnatal brain.

N-Cadherin (neural cadherin) is a transmembrane protein that mediates cell-cell adhesion and serves as a signal-transducing molecule and thus, is involved in the regulation of proliferation and differentiation (Chenn et al., 1998). N-Cadherin is localized in the apical parts of neuroepithelial cells and RGCs. In the adult SVZ, N-Cadherin expression is found in both quiescent and active Type B cells (NSCs) and in Type C cells (transient amplifying cells) (Klingener et al., 2014).

Dach1 (Dachshund homolog 1) is a chromatin-associated protein, its expression having been shown in neuroepithelial cells and RGCs of the VZ and SVZ of the developing neocortex, hippocampus and striatum (Machon et al., 2002; Castiglioni et al., 2018).

## Protocols

There are many protocols for obtaining NSCs from ESCs and iPSCs *in vitro* (Tables 1, 2). Mainly, these are based on the creation of specific conditions for the cultivation of stem cells and the addition of various molecules that affect their differentiation. With the accumulation of new data on neurogenesis, these methods have been shared, become more complicated and been combined with each other.

**TABLE 1:** Overview of the diversity of neural induction protocols (part 1).

		Method (2D/3D induction)	Cells	Protocols and conditions			Markers of NSC	Commitment	Self-renewal	Time*
1	Tropepe et al., 2001	(2D)	mESC	**mESC →** (Serum-free, low density, feeder layer free, FGF-2, LIF)	**→ floated colonies of NSC**		Nestin, Emx2, HoxB1	–	No	7 days
2	Kawasaki et al., 2000	SDIA (2D)	mESC	**mESC →** (Serum-free, low density, coculture on PA6, N2)	**→ NSC colonies**		Nestin, NCAM	Midbrain, hindbrain prog.	No	∼12 days
3	Perrier et al., 2004	SDIA (2D)	hESC	**hESC →** (Serum-free, low density, coculture on MS5)	**→ Neural rosettes**		Nestin, Pax6, Sox1, NCAM	Vent. midbrani, hindbrain (UCC)	No	28 days
4	Chambers et al., 2009	Dual SMAD inh. (2D)	hESC and hiPSC	**hESC / hiPSCs →** (Serum-free; SB; Noggin)	**→ early NSCs →** (Serum-free; SB; Noggin)	**→ Neural rosettes** (Serum-free; SB; Noggin)	Pax6, Sox1, Nestin, ZIC1	Anterior CNS	No	∼11 days
5	Patani et al., 2009	Monolayer (2D)	hESC	**hESC →** (Serum-free, feeder-free, low density, SB)	**→ NSCs** (Serum-free, SB, FGF-2)		Musashi-1, Sox1, Gbx2, HoxB6	Caudal CNS prog.	No	16 days
6	Li et al., 2011	pNSCs (2D)	hESC	**hESC →** (Serum-free, feeder-free, SB, CHIR, Compound E, hLIF, N2, B27)	**→ NSCs →** (Serum-free, feeder-free, SB, CHIR, Compound E, hLIF, N2, B27)	**→ NSCs expansion** (Serum-free, CHIR, hLIF, N2, B27)	Pax6, Sox2	Mesenceph. prog	Yes	7 days
7	Shi et al., 2012	Dual SMAD inh.+RA (2D)	hESC and hiPSC	**hESC / hiPSCs →** (serum-free, feeder-free, SB, Noggin, RA, N2, B27)	**→ early NSCs** (serum-free, feeder-free, SB, Noggin, RA, N2, B27)	**→ Neural rosettes** (serum-free, feeder-free, SB, Noggin, RA, N2, B27)	Pax6, FoxG1, Emx1, Otx1/2 N-cadherin, ZO-1	Telenceph. prog	No	15 days
8	Fedorova et al., 2019	(2D)	hESC and hiPSC	**hESC / hiPSCs →** (Serum-free, feeder-free, low density)	**→ NSCs →** (Serum-free, N2, B27)	**Neural rosettes** (Serum-free, N2, B27)	Pax6, Sox1, Sox2, PLZF, ZO-1, Dach1	–	No	8–10 days
9	Elkabetz et al., 2008	SFEB and SDIA (2D and 3D)	hESC	**hESC →EB → SFEB → plating →Neural rosettes →** (Serum-free, feeder free, Dkk1, LeftyA) **hESC →Neural →rosettes →** (Serum-free, coculture on MS5)	**→ isolation →R-NSC →** (Serum-free, FGF-2, EGF, FGF-8, SHH, AA, BDNF, N2) **→ isolation →R-NSC →** (Serum-free, FGF-2, EGF, FGF-8, SHH, AA, BDNF, N2)	**→NSC^FGF2/EGF^** (Serum-free, FGF-2, EGF, N2) **→NSC^FGF2/EGF^** (Serum-free, FGF-2, EGF, N2)	Nestin, Pax6, Sox1, Sox2, Musashi-1 (NSC^FGF2/EGF^ and R-NSCs) PLZF, ZO-1, Dach1, FoxG1 (R-NSCs) S100B and AQP (NSC^FGF2/EGF^)	Anterior CNS (dflt), caudal CNS and PNS fates (UCC)	R-NCS – limited NSC^FGF2/EGF^-yes	12-16 days (R-NSC)
10	Morizane et al., 2011	Dual SMAD inh.+SDIA or SFEB (2D and 3D)	hESC and hiPSC	**hESC / hiPSCs →** (Serum-free, coculture on PA6, SB, Dorsomorphin or Noggin) **hESC / hiPSCs →EB →SFEB→ plating→** (Serum-free, SB, Dorsomorphin or Noggin)	**→SFEB→ plating→** (Serum-free, coculture on PA6, SB, Dorsomorphin or Noggin) **→SFEB→ plating→** (Serum-free, SB, Dorsomorphin or Noggin)	**→Neural rosettes** **→Neural rosettes**	Nestin, Pax6, PSA-NCAM	Telenceph.prog (dflt), Ventr. midbrain prog. (UCC)	No	14 days

*Time required for NSC derivation from PSCs. (m/h)ESC, (mouse/human) embryonic stem cell; (h)iPSC, (human) induced pluripotent stem cell; EB, embryoid body; CNS, central nervous system; SFEB, serum free culture of embryoid bodies; SDIA, stromal-derived induction activity; Dual SMAD inh., dual SMAD inhibition; Telenceph., Telencephalic; Mesenceph., Mesencephalic; prog., progenitors; dflt, default; UCC, under certain conditions; RA, retinoic acid – (antagonist of β-catenin); PMA, purmorphamine (SHH stimulation); CHIR, CHIR99021 (GSK3 inhibitor); Dorsomorphin, Noggin (BMP inhibitors); SB, SB431542 and LeftyA (TGFβ/Activin/Nodal inhibitors); Dkk1, Dickkopf1 (Wnt antagonist); (h)LIF, (human) leukemia inhibitory factor; AA, ascorbic acid; Compound E (inhibitor of γ-secretase).

**TABLE 2:** Overview of the diversity of neural induction protocols (part 2).

		Method (2D/3D induction)	Cells	Protocols and conditions				Markers of NSC	Commit-ment	Self- renewal	Time*
11	Zhang et al., 2001	EB (3D)	hESC	**hESC → EB** (FGF-2)	**→ SFEB →** (FGF-2)	**→ plating → neural rosettes →** (FGF-2)	**→ NSC isolation → free-floating aggregates** (FGF-2)	Nestin, PSA-NCAM, Musashi-1	–	No	~14 days
12	Nakayama et al., 2004	NSS (3D)	mESC	**mESC →** (Astrocyte-conditioned medium, FGF-2, EGF)	**→NSS → plating →** (Serum-free, FGF-2, EGF, B27)	**→NSC colonies** (Serum-free, FGF-2, EGF, B27)		Nestin, Pax6	–	No	11 days
13	Watanabe et al., 2007	SFEB (3D)	mESC	**mESC → EB** (Serum-free, feeder-free, N2, B27)	**→ SFEB →** (Serum-free, feeder-free, N2, B27)	**→ plating → NSC colonies** (Serum-free, Dkk1, LeftyA, N2)		Nestin, Sox1, FoxG1	Telenceph. prog (dflt), midbrain, hindbrain (UCC)	No	~10 days
14	Eiraku et al., 2008	SFEBq (3D)	hESC and mESC	**mESC → quick EB formation→** (Serum-free) **hESC → quick EB formation →** (Serum-free)	**→SFEBq →** (Serum-free, Dkk1, LeftyA, N2) **→ SFEBq →** (Serum-free, Dkk1, LeftyA, BMPRIA-Fc)	**→SFEBq→** (Serum-free, N2) **→SFEBq→** (Serum-free, N2)	**→Dissociation and plating** (Serum-free FGF-2, N2) **→Dissociation and plating** (Serum-free FGF-2, N2)	Pax6, Sox1, N-cadherin, FoxG1, Emx1	Telenceph. prog (dflt)	No	10–12 days 46 days
15	Koch et al., 2009	SFEB-lt-hESNSC (3D)	hESC	**hESC →EB → SFEB →** (Serum-free, FGF-2)	**→ plating → neural rosettes** (Serum-free, FGF-2)	**→ NSC isolation → free-floating aggregates → dissociation** (Serum-free, FGF2, N2)	**→ plating in high cell density → rosettes-like colonies (lt-hESNSC)** (Serum-free, FGF-2, EGF, N2, B27)	Nestin, Pax6, Sox1, Sox2, Dach1, ZO-1, PLZF	Ventr. hindbrain (dflt); Ventr. midbrain (UCC)	Yes	28 days
16	Kim et al., 2011	Spin EB (3D)	hESC and hiPSC	**hESC / hiPSCs → EB** (Serum-free, Dorsomorphin or Noggin, N2, B27)	**→ SFEB → plating→** (Serum-free, Dorsomorphin or Noggin, N2, B27)	**→ Neural rosettes →** (Serum-free, FGF-2, Noggin, N2, B27)	**→ isolation and dissociation → NSCs monoculture** (Serum-free, FGF-2, EGF, Noggin)	Pax6, Sox1, Sox2, Dach1, NCAM, Emx2	Telenceph. prog.	–	7 days
17	Mariani et al., 2012	SFEBq (3D)	hiPSC	**hiPSCs → quick EB formation →** (Serum-free, FGF-2)	**→ SFEBq-like aggregates** (Serum-free, FGF-2, Dkk1, SB, BMPRIA-Fc)	**→ SFEBq-like aggregates** (Serum-free, Dkk1, SB, BMPRIA-Fc, N2)	**→ SFEBq-like aggregates** (Serum-free, B27)	Nestin, Pax6, Sox1, Sox2, N-Cadherin, Blbp	Telenceph. prog (dflt)	No	45–50 days
18	Kirkeby et al., 2012	(3D)	hESC	**hESC → EB →** (Serum-free, SB, Noggin, CT99021, SHH-C24II, N2, B27)	**→ SFEB → plating →** (Serum-free, SB, Noggin, CT99021, SHH-C24II, N2, B27)	**→ clusters of NSCs →** (Serum-free, SB, Noggin, CT99021, SHH-C24II, N2, B27)	**→ Dissociation and plating** (Serum-free N2, B27)	FoxG1, SIX3, NKX2.1	Telenceph. prog (dflt), hindbrain, midbrain (UCC)	No	11–14 days
19	Reinhardt et al., 2013	smNPC (3D)	hESC	**hESC → EB→** (Serum-free, SB, Dorsomorphin, CHIR, PMA, N2, B27)	**→EB→ plating→** (serum-free, CHIR, PMA, AA, N2, B27)	**→ smNPC →** (serum-free, CHIR, PMA, AA, N2, B27)	**→ Neural rosettes** (serum-free, FGF-2, CHIR, PMA, AA, N2, B27)	Nestin, Pax6, Sox1, Sox2, Dach1, PLZF (smNPC), N-cadherin, ZO-1 (rosettes)	Neural crest and posterior CNS (UCC)	Yes	16 days
20	Rosati et al., 2018	hiNSC (3D)	hiPSC	**hiPSCs → EB →** (Serum-free)	**→EB →** (Serum-free; O_2_ 20%)	**→ EB →** (Serum-free)	**→ EB → dissociation → Neurosphere → expansion → plating → hiNSCs culture** (Serum-free, FGF-2, EGF)	Pax6, Sox2, GLAST, GFAP, BLBP	–	Yes	6–8 days

*Time required for NSC derivation from PSC. (m/h)ESC, (mouse/human) embryonic stem cell; (h)iPSC, (human) induced pluripotent stem cell; EB, embryoid body; NSS, neural stem spheres; CNS, central nervous system; SFEB(q), (quick) serum free culture of embryoid bodies; Telenceph. Prog, Telencephalic progenitors; dflt, default; UCC, under certain conditions; PMA, purmorphamine (SHH stimulation); CHIR, CHIR99021 (GSK3 inhibitor); Dorsomorphin, Noggin and BMPRIA-Fc (BMP inhibitors); SB, SB431542 and LeftyA (TGFβ/Activin/Nodal inhibitors); Dkk1, Dickkopf1 (Wnt antagonist); AA, ascorbic acid.

**FIGURE 1:**
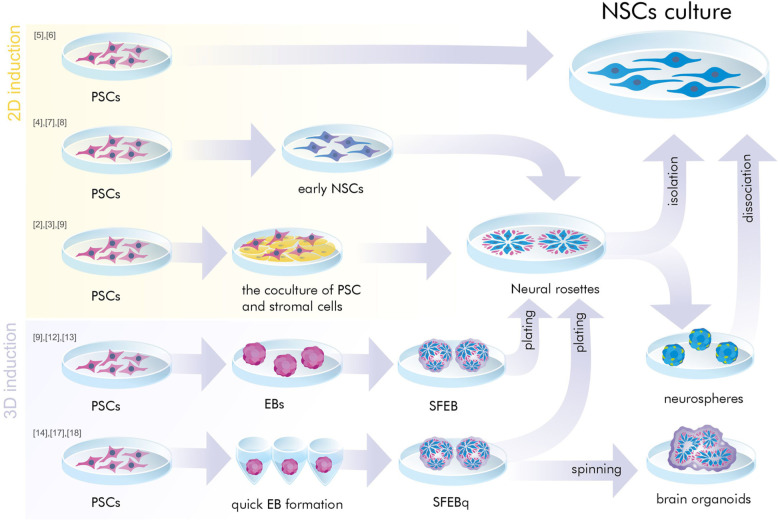
A simplified presentation of the general principles of neural induction. Neural induction of PSCs may be conducted in 2D conditions (monolayer and coculture) or in 3D conditions (embryoid bodies). At the same time, further generation and/or cultivation of NSCs can be achieved in 2D monoculture and neural rosettes or in 3D cultures (SFEB and SFEBq, brain organoids, neurospheres). PSCs, pluripotent stem cells; NSCs, neural stem cells; EB, embryoid body; SFEB(q), (quick) serum free culture of embryoid bodies; [№] are references to examples of the protocols presented in Tables 1, 2.

Neural induction of PSCs may be conducted in 2D conditions (monolayer) (Chambers et al., 2009; Patani et al., 2009; Fedorova et al., 2019) or in 3D conditions (embryoid bodies) (Figure 1) (Watanabe et al., 2007; Eiraku et al., 2008). At the same time, further generation and cultivation of NSCs can be achieved in several ways: 2D monoculture (Patani et al., 2009; Fedorova et al., 2019), 2D neural rosettes (Watanabe et al., 2007; Elkabetz et al., 2008; Chambers et al., 2009), through 3D cultures (Eiraku et al., 2008; Mariani et al., 2012), or in organoids (Lancaster et al., 2013; Pasxca et al., 2015; Qian et al., 2016).

### Default Differentiation Protocols

Prolonged disaggregation of Xenopus germinal ectoderm cells in the absence of a tissue organizer led to the expression of neural markers by these cells (Grunz and Tacke, 1989). Embryonic stem cells transplanted into the inner cell mass of an embryo, as well as intrinsic cells of the inner cell mass, were included in the formation of the neuroectoderm (Gossler et al., 1989). This means that PSCs acquired a neural fate without exogenous instructions, and this fate could be supported by blocking neural induction inhibitors. In 1995 Bain et al. managed to differentiate neuron-like cells from embryoid bodies (EBs) cultured in a medium supplemented with RA, an antagonist of β-catenin (Bain et al., 1995). Later it turned out that RA causes differentiation into neural cells with caudal specification and blocks the differentiation of neurons of the central part of the CNS (Wichterle et al., 2002).

Based on this default-like mechanism of neural tissue formation, Tropepe et al. (2001) obtained floating spheres (neurospheres) of primitive NSCs from single ESCs of mice (mESCs), on chemically defined serum-free medium, feeder layer-free, in low-density culture conditions, in the presence of leukemia inhibitory factor (LIF) and FGF-2. Such floating colonies of NSCs expressed Nestin, possessed several traits of the NSCs of the forebrain and were able to generate neurons (MAP2+ or βIII-tubulin+), astrocytes (GFAP+), and oligodendrocytes (O4+) over a period of 7 days after dissociation. The authors showed that low-density ESCs have an autonomous tendency for neural differentiation that decreases with increasing cell density and cellular interactions. Furthermore, they proposed that differentiation of the ESCs at high densities into NSCs may be more effective in the presence of the TGF-β-related signaling inhibitors (Chordin and Noggin), due to the negative effect of BMPs on the neuronal specification (Wilson and Hemmati-Brivanlou, 1995; Tropepe et al., 2001). Later, the suggestion regarding the inhibition of TGF-β-signaling was supported by experiments to obtain NSCs from hESCs cultured on serum-free medium in the presence of Noggin (Pera et al., 2004; Itsykson et al., 2005).

The need for the absence of serum in the medium was also confirmed by studies with Sox1-GFP knock-in ESCs. Sox1 is the earliest marker of mouse neuroectoderm. Its expression shows up only in serum-free culture medium and in the absence of LIF (Ying et al., 2003). They also found that the addition of N2 and B27 supplements to serum-free culture media increased the efficiency of neural differentiation. Several experiments have demonstrated neural differentiation of ESCs and iPSCs using monolayer culture in the presence of N2 and B27, with (Ying et al., 2003; Lin et al., 2010; Matulka et al., 2013), and without FGF-2 (Lippmann et al., 2014; Fedorova et al., 2019).

### Neural Stem Spheres

Previously it had been shown that neuroepithelial progenitor isolated from the embryonic brain could be grown *in vitro* as free-floating spherical aggregates called “neurospheres,” consisting of NSCs, differentiated neurons and astroglial cells (Reynolds et al., 1992; Buc-Caron, 1995). Nakayama et al. (2004) proposed a method for producing NSCs from mESCs via the neurosphere-like aggregates. They showed that mESC colonies, cultured in non-adhesive dishes with astrocyte-conditioned medium, FGF-2, and EGF, were able form neural stem spheres (NSSs) that consisted of an outer layer of the Nestin-positive NSCs surrounding a core of mESCs. After culturing the NSSs on an adhesive substrate with mitogens, the NSCs migrated onto the substrate and formed a monolayer culture that could be differentiated into neurons (Nakayama et al., 2004).

### Neural Rosettes

The next historical step was to obtain NSCs from ESCs through neural rosettes. Neural rosettes are self-organized clusters of polarized neuroepithelial-like cells, resembling an embryonic neural tube. The radially organized cells of these structures can create a lumen, similar to the VZ of a developing brain (Elkabetz et al., 2008; Ziv et al., 2015). Cells of such clusters exhibit the morphological signs of early neuroepithelial cells and RGCs and express the neuroectodermal markers Pax6, Sox1, Sox2, NCAM, Nestin, and Musashi-1. Neural rosettes are capable of differentiating into more committed neural precursors that can then generate distinct neurons types and glia. Forebrain fate is the default for the NSCs of neural rosettes, however they have a differentiation potential toward both CNS and peripheral nervous system (PNS) fates and they can generate midbrain, striatal, spinal cord or even neural crest neurons when different morphogens are used (Perrier et al., 2004; Elkabetz et al., 2008; Koch et al., 2009). Clusters of neural rosettes can be isolated and maintained *in vitro* as a culture of NSCs. In 2001, Zhang et al. (2001) showed that hESCs in the form of EBs could be directed toward neural precursor cells. To generate the EBs, hESCs were grown in suspension for 4 days. The resulting EBs were cultivated on adhesive substrate in the presence of FGF-2. Over a period of 7 days in these conditions, the plated EBs started to generate neuroepithelial cell self-organized clusters – neural rosettes that expressed the neural markers Nestin, PSA-NCAM and Musashi-1. However, besides NSCs, there were cells with markers characteristic of neurons, glia and hESCs in the culture. The authors then isolated the neural precursors and expanded them as free-floating cell aggregates (neurospheres) with FGF-2. After plating of these aggregates on ornithine and laminin substrate, they were capable of generating the three CNS cell types (Zhang et al., 2001).

### Stromal Feeder-Based Differentiation Protocols and Stromal Derived Induction Activity

Using the stromal feeder-based differentiation approach [stromal derived induction activity (SDIA)-mediated protocols) the differentiation of neuroepithelial cells from hESCs has been achieved by their cocultivation on MS5 or PA6 stromal cells in serum-free medium. MS5 and PA6 are stromal cell lines, isolated from bone marrow, that are used as a feeder layer for maintaining the growth of hematopoietic stem cells (Itoh et al., 1989). It was shown that these stromal cell lines exhibit neural-inducing properties during cocultivation with ESCs (Kawasaki et al., 2000, 2002). The mechanism of such induction is still unknown. This protocol allowed typical neural rosettes to be obtained expressing NSC markers (Pax6, Sox1, Nestin, and NCAM) without needing EB formation (Kawasaki et al., 2000; Perrier et al., 2004; Elkabetz et al., 2008). However, not all the ESCs differentiate into neuronal precursors under the described conditions, a proportion of the cells differentiate into non-neural cells while 10–15% of the cells remain in an ESC state (Ying et al., 2003). It has been shown, that SDIA-induced neural precursors more frequently expressed markers of the midbrain and hindbrain (Kawasaki et al., 2000; Watanabe et al., 2007).

### Serum Free Culture of Embryoid Bodies

In 2000 Lee et al. (2000) created a protocol for NSC generation from mouse ESCs using an EB-formation stage. To achieve this, formed mEBs were plated and then cultured with serum-free Insulin/Transferrin/Selenium/Fibronectin medium for 6 days. Next, the culture was expanded in the presence of bFGF and laminin, either in the presence or absence of a combination of FGF8 and SHH, for 8 days. The resulting NSC culture was prone to differentiation into midbrain and hindbrain neurons.

Subsequently, Watanabe et al. modified this method using EB-formation, and were able to obtain NSCs through neural rosettes from mouse EBs cultured in serum-free culture medium. The method was named serum free culture of embryoid bodies (SFEB; Watanabe et al., 2005). In addition, they showed that treatment of the EB cultures with Dickkopf1 (Dkk1) (a Wnt antagonist) and LeftyA (TGF-β/Nodal/Activin antagonist) achieved 90% efficiency of differentiation of the mESCs into NSCs (Watanabe et al., 2005). They also showed that the addition of Wnt3a, Nodal or BMP4 suppressed neural conversion, confirming the inhibitory effect of Wnt- TGF-β- and BMP-signaling on neural induction (Aubert et al., 2002; Vallier et al., 2004; Liu and Niswander, 2005). In 2007, the same approach, but with the application of a selective Rho-associated kinase (ROCK) inhibitor, was applied to hESCs (Watanabe et al., 2007).

### Dual-SMAD-Inhibition

A significant breakthrough was made in 2009 by Chambers et al. (2009). Their approach made it possible to obtain neuroepithelial cells from hESCs and hiPSCs with high efficiency and without any EB cultivation stage or cocultures with stromal cells. This method is based on suppression, using Noggin, of two main signaling pathways prohibiting the neural differentiation of pluripotent cells: the TGF-β/Activin/Nodal pathway via SB431542 and the BMP-canonical pathway (Chambers et al., 2009). SB431542 inhibits the Activin type I receptor, thus preventing Smad2/3-signaling (Smith et al., 2008). Noggin inactivates BMP4, causing cancelation of Smad1/5/8-signaling (and also of MAPK p38) (Pera et al., 2004). This is why the protocol has been termed Dual-SMAD-inhibition. In this approach the researchers cultured hPSCs onto Matrigel-coated dishes in serum-free culture media with the addition of SB431542 and Noggin. After a 5-day exposure of the hPSCs to Noggin/SB431542, the cells became a Sox1-, Pax6- and ZIC1-positive early neuroepithelial population capable of neural rosette organization (Chambers et al., 2009). This protocol showed an 80% efficiency of hESC and hiPSC differentiation into Pax6-positive NSCs.

The combination of Dual-SMAD-inhibition with the SDIA and SFEB methods was also used by Morizane et al. (2011). However, unlike the original method, they used Dorsomorphin as an alternative to Noggin. Several lines of hESCs and iPSCs were cultivated on PA6 stromal feeder cells or as an SFEB culture. They were able to obtain neural rosettes of NSCs with high efficiency and found that Dorsomorphin works better than Noggin and increases the survival of the colonies (Morizane et al., 2011). Dorsomorphin is a small molecule, which, unlike Noggin, selectively inhibits Smad1/5/8 (but not MAPK p38). It is cheaper than Noggin (Noggin is a recombinant protein), has less variability between batches and is safer for clinical trials (Yu et al., 2008). Comparing the spontaneous differentiation of EBs of different lines of hESCs and hiPSCs with each other and with induced differentiation, it was found that treatment with SB431542 and Dorsomorphin during the spontaneous differentiation of EBs leads to more efficient neural differentiation and alignment of the differences in propensity for differentiation in the different lines of hPSCs (Morizane et al., 2011). Addition of RA to the Dual-SMAD inhibition protocol significantly improved the efficiency (>95%) of hESC and hiPSC differentiation into Pax6-positive cortical NSCs and progenitor cells (Shi et al., 2012).

Kim et al. (2010) modified the SFEB protocol: they used fast aggregation of hESCs or hiPSCs in EBs [as in the SFEBq method (see below)] in the presence of Noggin and termed it the Spin EB method. After the EBs were seeded onto an adhesive substrate, they generated neural rosettes. It was found that BMP inhibition was not required to initiate formation of the rosettes but did influence their morphology: the absence of Noggin led to the formation of defective rosettes and cell flattening. However, rosettes obtained with the spin EB method exhibited typical features: neuroepithelial cell morphology, and expression of the markers of neural progenitors: Sox1, Sox2, Pax6, Dach1, and NCAM. Rosette dissociation and passaging in the presence of FGF-2 and EGF gave rise to monocultures of neural progenitors capable of differentiating, by default, into dorsal forebrain neurons with formed synaptic connections (Kim et al., 2010).

An analysis of the development of hiPSCs and ESCs exposed to Dual-SMAD inhibition has shown, that the neural rosettes repeat the developmental stages of the cerebral cortex in humans. An interkinetic nuclear migration process is distinctive for neuroepithelial stem cells of the VZ and SVZ of the brain (Shi et al., 2012; Grabiec et al., 2016). Rosette populations were capable of generating three types of cortical stem/progenitors: neuroepithelial cells, RG-like cells and IPCs. This *in vitro* system also recapitulated the temporal order of the genesis of projection neurons corresponding to that during *in vivo* neurogenesis (Shi et al., 2012).

Elkabetz et al. provided a more detailed description of the properties of neural rosettes obtained by both SDIA-based neural induction and the SFEB protocol (Perrier et al., 2004; Watanabe et al., 2007; Elkabetz et al., 2008). Early neural rosettes can be heterogeneous and contain not only neural progenitors, but also differentiated neurons, non-neural derivatives and undifferentiated ESCs. The cells of neural rosettes have the competence to generate region-specific neuronal and glial cell types under certain conditions. The NSCs of neural rosettes (R-NSCs) proliferate in the presence of FGF-2 and EGF and give rise to more restricted NSC-like cells (NSC^FGF–2/EGF^s). Both R-NSCs and NSC^FGF–2/EGF^s express common NSC markers such as Pax6, Sox1, Sox2, Musashi-1 and Nestin. However, R-NSCs are characterized by additional expression of specific markers including PLZF, ZO-1, and Dach1, while NSC^FGF–2/EGF^s show the expression of the later stage neural precursor markers – S100β and AQP (Elkabetz et al., 2008). They showed that NSC^FGF–2/EGF^s are very similar to symmetrically dividing NSC populations, while R-NSCs correspond to the neural plate stage. Furthermore, the transplantation of R-NSCs into adult rodent brain showed neural overgrowth of the transplanted grafts, and that this effect was not associated with hESC contamination (Elkabetz et al., 2008). These data suggest that NSC^FGF–2/EGF^s are the next and more restricted stage of development of R-NSCs that inevitably occurs. Maintenance the R-NSC state depended on the cell density and on Notch-signaling: high cell densities in the presence of SHH/FGF-8 maintained the neural rosettes, while low plating densities led to enhanced neural differentiation (Elkabetz et al., 2008; Edri et al., 2015).

However, neither NSC^FGF–2/EGF^s nor R-NSCs are a stable source of NSCs. Subsequent expansion of NSC^FGF–2/EGF^s both changes their differentiation potential and increases their resistance to *in vitro* regionalization (Elkabetz et al., 2008). While R-NSCs do have an increased differentiation potential, they can only be expanded during 4 passages without losing this potential (Elkabetz et al., 2008). Long-term cultivation of neural rosettes led to a change in the morphology of cellular composition and their potential for differentiation. Initially, R-NSCs contain highly proliferative NSCs with a wide differentiation potential, but after a while they progress into more heterogeneous rosettes with decreased numbers of NSCs and a tendency to differentiate into neurons. Later still, the neural rosettes begin to lose their epithelial integrity and rosette organization and are characterized by low NSC numbers, increased cellular heterogeneity and a tendency to differentiate into glial cells (Edri et al., 2015). Considering these restrictions, Koch et al. set a goal of obtaining a source of stable and self-renewing NSCs. They carefully isolated EB-derived neuroepithelial cells from neural rosettes and cultivated them as floating spheres (neurospheres) for 24 h. After that the spheres were dissociated and plated as a monolayer in high-cell density conditions. Such cultures came to form rosette-like patterns. In this way they managed to obtain homogeneous populations of neural progenitors that partially exhibited the morphological properties and expression patterns of early R-NSCs (Elkabetz et al., 2008). These neural progenitors could be extensively propagated through more than 150 passages in the presence of FGF-2 and EGF without losing their progenitor characteristics and could be differentiated into different neuronal types using defined morphogens. Thus Koch et al. (2009) had created long-term, self-renewing NSCs (lt-hESNSCs).

Subsequently there was an attempt to create long-term self-renewing NSCs from hESCs without the EB stage. Exposure of hESCs to CHIR99021, SB431542, Compound E and hLIF in a serum-free culture medium for 7 days induced their differentiation into primitive neuroepithelium. Such primitive NSCs (pNSCs) could be stably expanded as a homogeneous population under hLIF/CHIR99021/SB431542 conditions without loss of their differentiation potential or proliferative capacity (Li et al., 2011). CHIR99021 inhibits glycogen synthase kinase 3 (GSK3) and therefore activates Wnt/β-catenin-signaling (Sato et al., 2004). Compound E is a small molecule, an inhibitor of γ-secretase, which is involved in Notch-signaling (Li et al., 2011). Primitive NSCs s correspond to a pre-rosette state of NSCs that can organize into neural rosettes after being cultured in the presence of FGF-2. There is a similarity between pNSCs and NSC-populations generated via the Dual-SMAD inhibition protocol, however, the second type represents a heterogeneous neural population of primitive neuroepithelial and polarized rosette-forming cells, the proliferative and differentiation potentials of which cannot be maintained in culture. In addition, the new method takes only 7 days to obtain NSCs from hESCs in contrast to the 13-day Dual-SMAD inhibition protocol (Chambers et al., 2009; Li et al., 2011). Primitive NSCs can be effectively differentiated into different neuron types under appropriate conditions (Li et al., 2011).

Despite the variety of protocols for obtaining NSCs using various morphogens and inhibitors (Zhang et al., 2001; Perrier et al., 2004; Watanabe et al., 2007; Chambers et al., 2009; Koch et al., 2009; Li et al., 2011), it has turned out that they are not necessary for the successful initiation of neural differentiation of PSCs. Based on the 2D culture system, a protocol with a high efficiency (>95%) of NSC generation from hiPSCs in a serum-free culture medium, with low cell density plating, and without any morphogens or factors (FGF-2, BMP-inhibitors and others) was developed (Fedorova et al., 2019). This method enables obtaining a homogenous Pax6/Sox1 positive population of NSCs in 6 days of hiPCS cultivation in the differentiation medium. By the 8–10th days of the differentiation protocol, such NSCs have formed neural rosettes, although these spontaneously regress by the 14th day. These neural rosettes, as in the other protocols, expressed ZO-1, PLZF, and Dach1 (Fedorova et al., 2019). Surprisingly, extra inhibition of BMP-signaling by the addition of inhibitors was not required to obtain NSCs in this protocol. There were no significant changes in the expression of pluripotency- or neuro-specific-markers [except for Sox1, Sox2 (increased) and Pax6 (reduced)] in the presence of BMP inhibitor, so this suggests that BMP might be active during the development of neural rosettes. Moreover, BMP may be involved in the formation of the 3D organization of the neural rosette. However, the addition of the BMP inhibitor to the differentiating medium did cause changes in the morphology of the neural rosettes: they were small and flat, in contrast to the large rosettes with specific “ridges” present in the standard conditions (Fedorova et al., 2019). These data contradict those obtained with previous protocols (Chambers et al., 2009; Kim et al., 2010; Li et al., 2011), although, as suggested by the authors of this study, low cell density may be a decisive parameter for the initiation of neural induction in the absence of inhibitors (Fedorova et al., 2019).

It has been suggested that neural rosette organization *in vitro* mimics primary neurulation *in vivo* (Grabiec et al., 2016), however Fedorova et al. compared it with secondary neurulation *in vivo*, based on the observation that the lumens of smaller rosettes fuse to form one and that molecular markers associated with secondary neurulation were expressed during this process (Fedorova et al., 2019).

Reinhardt et al. (2013) set themselves the task of obtaining NSCs that were able to differentiate not only into the neurons of the forebrain but also into other neural tube and neural crest lineages. They exposed EBs from hESCs to SB431542 (TGF-β/Activin/Nodal inhibitor), Dorsomorphin (BMP inhibitor), CHIR99021 (GSK3 inhibitor), and purmorphamine (PMA; Reinhardt et al., 2013). Purmorphamine provides SHH stimulation, which is known to direct ventral neural tube fates (Sinha and Chen, 2006). After 4 days of neural induction, the SB431542 and Dorsomorphin were withdrawn and ascorbic acid was added. Differentiating EBs began to express neuroepithelial markers – Sox1, Sox2, and Pax6. Disaggregated and plated EBs formed homogeneous colonies of neural progenitor cells (smNPCs) expressing markers of early neural progenitors. These smNPCs corresponded to pre-rosette NSCs and could be expanded through up to 20 passagings. Exposure of the smNPCs to FGF-2 resulted in the formation of typical neural rosettes. Cultivation of the smNPCs with only CHIR99021 led to neural crest fate specification; with only PMA – to posterior CNS fate specification; and with PAM and FGF-8 – to form midbrain neurons. Thus, it was shown, that CHIR99021 and PMA are able to direct the differentiation of neural progenitors toward ventral neural tube- and neural crest-derived lineages respectively (Reinhardt et al., 2013).

For use in clinical trials of cell therapy, it is necessary that hiPSC-derived NSCs should form a stable homogeneous population with well-defined characteristics of NSCs, with predictable behavior, and be safe (from the point of view of tumorigenic ability). Rosati et al. (2018) obtained a hiPSC-derived NSC line (hiNSCs) that had characteristics similar to those of GMP certified fetal hNSCs that have been approved for clinical applications by the Italian Medicines Agency (AIFA, aM 154/2018). This protocol of neural induction differs from the others in its duration (2 months for NSC generation, and 4–8 months for expanding) and the absence of a need for exposure to morphogens. Neural induction was achieved by prolonged hiPSC-derived EB cultivation under serum-free conditions (63 days) followed by dissociation and amplification as hiNSCs neurospheres, originating from individual cells (Rosati et al., 2018). The long amplification period allowed degeneration a greater number of differentiated types of NSCs (transient amplifying cells) from the resulting heterogeneous culture, meaning that a stable homogeneous line of multipotent NSCs could be obtained. The derived hiNSCs expressed markers of RGCs, were capable of extensive self-renewal and of differentiation into neuronal, astroglial, and oligodendroglial lineages *in vitro* (Rosati et al., 2018).

Most of the protocols described above have made it possible to obtain heterogeneous populations of NSCs including other types of cells (Zhang et al., 2001; Perrier et al., 2004; Watanabe et al., 2007; Chambers et al., 2009). In addition to NSCs, such populations could include undifferentiated iPSCs and their random derivatives plus intermediate differentiation variants of NSCs, as well as neurons and glia cells. Residual iPSCs in NSC cultures can distort further experiments with such cultures, since they can affect the differentiation process and the quality of subsequent neuronal cultures, alter the effects of drug tests and cause tumors after transplantation when used as part of cell therapy.

To date, various methods have been developed to remove pluripotent cells remaining in target cultures after differentiation: microfluidic separation, PSC elimination with cytotoxic antibodies and small molecules, photoablation-based cell-depletion, magnetic activated cell sorting (MACS), affinity chromatography etc. The advantages and disadvantages of various strategies for such cell purification to remove PSCs were reviewed in detail by Rodrigues et al. (2015a, b). In particular, MACS was applied for the purification of hPSC-derived NSCs from cultures including remaining hPSCs (Rodrigues et al., 2014, 2015a). However, sometimes, removing only PSCs from a heterogeneous culture of NSCs is insufficient. To isolate a pure population, positive selection of the NSCs is necessary. Methods such as fluorescence-activated cell sorting (FACS) and MACS may be suitable for this purpose. Yuan et al. (2011) made an attempt to develop a set of FACS identifiers. They selected 4 cell surface antigens (CD184^+^/CD271^–^/CD44^–^/CD24^+^) that enabled isolation of the NSCs from heterogeneous cultures of neural rosettes obtained from hiPSCs and hESCs using various methods (SFEB, SDIA, Dual-SMAD inhibition). Unfortunately, this set of antigens was able to select only one of the NSC subpopulations present in the heterogeneous cultures after neural induction. Therefore, further research is necessary to develop a more complete set of NSC markers, allowing them to be used to select all types of NSC subpopulation. Despite its high efficiency, FACS has some disadvantages, in particular, it can reduce the viability of the isolated cells. In this regard, MACS has advantages, since the selection and isolation of the cells using magnetic nanoparticles coated with antibodies is less traumatic. Therefore, Bowles et al. (2019) developed an efficient two-step MACS protocol to isolate CD271-/CD133 + NSC population.

### SFEBq

The SFEB method has been modified, involving three-dimensional aggregates of differentiating EBs that are called SFEBqs. Such controlled, quick formation of embryonic bodies with uniformity of size resulting from the use of wells with U-shaped bottoms, allows an increase in the rate and efficiency (>95%) of production of neural precursors (Eiraku et al., 2008). Mouse EBs were cultivated in serum-free medium with the addition of Dkk1 and LeftyA, while human EBs were cultured with the addition of Dkk1, LeftyA and human, soluble BMPRIA-Fc (as a BMP inhibitor). Under such conditions, neuroepithelial rosette-like structures began to form in the ESC aggregates. After dissociation of the SFEBq culture the neural progenitors migrated to the adhesion substrate and differentiated into cortical-type neurons. This model therefore supports cortical neuroepithelium formation in a three dimensional, spatially and temporally controlled pattern (Eiraku et al., 2008). Subsequently, this protocol was adapted for hiPSCs (Mariani et al., 2012). Such aggregates represent multilayered structures of neural progenitors with an apical-basal arrangement, within which cells form neural tube-like substructures (3D rosettes). The neural progenitors of such rosettes exhibit expression of the neuroepithelial markers Sox1, N-cadherin and FoxG1 (Eiraku et al., 2008; Mariani et al., 2012). The hiPSC-derived 3D aggregates reflect the *in vivo* cytoarchitecture of developing human brain and include NSCs, IPCs and neurons showing synapse formation. The transcriptome of these structures cultivated for 50 days was similar to that of the developing human cerebral cortex of the 8–10th week (Mariani et al., 2012). Thus, this group had managed to partially recreate the early development of the human dorsal telencephalon in a 3D system *in vitro.*

Based on a similar approach, 3D culture systems for PSC-derived neural tissue cultivation have been developed (Lancaster et al., 2013; Pasxca et al., 2015; Qian et al., 2016). Brain tissue organoids can be obtained from hEBs cultured with (Lancaster et al., 2013) or without (spontaneous organoid differentiation) (Pasxca et al., 2015), the use of morphogens. In the early stages, such aggregates consist of neuroepithelium surrounding ventricular-like cavities and exhibit neuroepithelial markers. On reaching a certain size, the spontaneously differentiated organoids gain increased heterogeneity, where there are regions resembling various brain tissues: cerebral cortex, choroid plexus, retina, and meninges. The controlled conditions of differentiation make it possible to obtain specific structures called “spheroids” corresponding to certain areas of the brain: the cerebral cortex, hippocampus, and midbrain (Qian et al., 2016, 2018). For this, different induction protocols can be used. For example, the application of the Dual-SMAD inhibition protocol for forming EBs from iPSCs allows the generation of cortical spheroids, which can be maintained in neurobasal medium containing FGF-2, EGF, and B27 supplement (Pasxca et al., 2015). Such floating spheroids consist of Pax6 and FoxG1-expressing neural progenitors that are able to differentiate into cortical neurons as well as astrocytes after 7–8 weeks in culture with brain-derived neurotrophic factor (BDNF) and neurotrophic factor 3 (NT3). Such cortical spheroids resemble the mid-fetal prenatal brain (Pasxca et al., 2015). Three-dimensional brain organoids recreate a more physiologically natural environment for neuronal development and interactions with other neurons, as well as with astrocytes and the extracellular matrix. However, these organoids have several limitations. For example, during the later stages of organoid cultivation, due to the absence of vasculature and their large size, the deeper layers lack nutrients and oxygenation. Therefore, a necrotic core is formed. The issue is that brain organoids are able to generate all types of cells of the neuroectodermal line, including various types of neurons, astrocytes, and oligodendrocytes, but are not able to form endothelial cells. In search of a solution to this problem, Mansour et al. (2018) proposed a method of intracerebral implantation of brain organoids grown *in vitro* into the brains of mice. The implanted organoids were invaded by blood vessels from the host brain and they not only survived, but also actively differentiated, with the neurons showing axonal growth and even the formation of synapses with host neurons. Pham et al. made an attempt to create organoids with their own vessels and implant them in the brains of mice. For this they added exogenous endothelial cells to the organoids at the EB stage, unfortunately, they found that the resulting primitive tubular network could not provide a complete supply of nutrients to the deep layers of the organoids *in vitro*. In an attempt to resolve this, the vascularized organoids were transplanted into immunodeficient mice (Pham et al., 2018). However, they did not prove connectivity of the organoid’s and host’s capillaries. Co-culture of hiPSC with human umbilical vein endothelial cells followed by the formation of EB and neural induction has provided the development of cerebral organoids with tube-like vascular systems (vOrganoids). Transplanted into mouse brains, such vOrganoids integrated into the host’s brain, and the blood vessels of the transplant and recipient became connected (Shi et al., 2020). Organoid vascularization can be achieved by the fusion of iPSC-derived early brain spheroids (composed of NSCs) and iPSC-derived vascular spheroids (composed of endothelial cells) in the presence of human mesenchymal stem cells (Song et al., 2019). Another approach to solving the problem has been to induce the differentiation, within the organoid, of its own vessels. For this, after EB formation combined with simultaneous neural induction, vascular endothelial growth factor (VEGF) and Wnt7a were added to the differentiating medium. This method allowed the generation of cerebral organoids with vascular structures, without inhibiting neuronal differentiation (Ham et al., 2020).

## Discussion

Protocols for obtaining NSCs *in vitro* have been developing since the end of the 20th century. Unsurprisingly, due to insufficient data on the detailed development of the nervous system and the molecular mechanisms of differentiation of NSCs, the first protocols had significant drawbacks and low efficiency. However, the subsequent development of this area, and the approaches to obtaining NSCs have been repeatedly improved. Nevertheless, it is very difficult to compare the efficiencies of the protocols for NSC differentiation from PSCs due to the use of (1) different lines of PSCs; (2) different methods and markers for NSC determination (3) different methods for calculating the effectiveness of differentiation; and (4) the different purposes for which the NSCs have been obtained. In addition, the authors have often only subjectively evaluated the efficiency of differentiation. Using a protocol for mESCs with a serum-free medium and low cell concentration, Tropepe et al. (2001) achieved only 24% of Nestin-positive cells out of the total number of ESCs after 24 h (of which 70% did not survive). In the same conditions Fedorova et al. were able to achieve 95% NSC differentiation from iPSCs, however, they used Pax6 and Sox1 to identify the NSCs (Fedorova et al., 2019). The original SFEB protocol could provide 90% of cells with Sox1 expression by day 5 of mEB culture, but only 35% of the cells were Bf1-positive (Watanabe et al., 2005), while 65–75% of the cells were Bf1-positive using the SFEBq modification (Eiraku et al., 2011). The dual SMAD inhibition protocol developed by Chambers et al. has an 80% efficiency of PSC differentiation into Pax6-positive NSCs (Chambers et al., 2009). The combination of Dual SMAD inhibition with RA to form ESCs showed a 95% yield of Pax6, Oct1/2, vimentin-positive cells (Shi et al., 2012). Despite the abundance of neural induction protocols, by far the most popular is Dual SMAD inhibition. Although it does not provide the highest efficiency, and it results in a rather heterogeneous culture, this method has proven to be the most reliable and easy to use. This protocol is easily modified and used for both the 2D and 3D induction of NSC.

Each of the 2D and 3D approaches to neural induction has advantages and disadvantages. 2D induction usually takes less time than 3D induction via EBs, is easier to perform and has a greater efficiency (Yan et al., 2013). 3D induction, by contrast, presents technical difficulties and requires longer. Although both methods can provide proper rosette formation and differentiation of the derived NSCs into mature neurons, 3D induction shows several advantages. Comparison of Dual-SMAD inhibition (2D neural induction) with a combination of Dual-SMAD inhibition and SFEB protocols (3D induction) revealed that the 3D induction may promote greater numbers of NSCs with a high level of expression of Pax6 and Nestin, resulting in more forebrain neurons and a gain in neurite outgrowth (Chandrasekaran et al., 2017). This suggests that three-dimensional signal transmission and cell–cell interactions are probably also important for neuronal induction. This research compared 2D and 3D induction in an example with only one protocol that used distinct morphogens, however there are many other different protocols and methods available to obtain NSCs from PSCs.

Different methods of further cultivation are also used during PSC differentiation: monolayer culture, neural rosettes, 3D aggregates, and organoids. Monolayer culture is the easiest option for generating NSCs as it presents the least technical difficulties and does not require special equipment. With this approach, it is easiest to obtain a homogeneous population of identical NSCs. However, there are not many such protocols. It is considered that this type of NSC culture has limited differentiation potential, although some reports have shown that the NSCs obtained in this way still have a fairly wide potential (Yan et al., 2013). The next step of complexity is the generation of NSCs via neural rosettes. A neural rosette is not quite a 2D culture, but neither is it 3D. The cells of neural rosettes are polarized and organized into complex structures with special cell-cell interactions that better recapitulate the development of the neural system (Elkabetz et al., 2008; Ziv et al., 2015; Fedorova et al., 2019). Such rosette structures can be compared to the neural tube. One group has proposed that the process of rosette formation mimics secondary neurulation *in vivo* (Fedorova et al., 2019). Despite this advantage, the neural rosette approach has limitations. Neural rosettes often represent heterogeneous populations; therefore, additional manipulations are required to isolate pure lines of NSCs. The EB-derivation of rosettes (SFEB method) (Watanabe et al., 2007) is more difficult, as it requires special conditions and equipment, unlike the 2D rosette generation approaches such as Dual-SMAD inhibition (Chambers et al., 2009). There are many different protocols that are able to provide the generation of rosettes of neural progenitors. The protocols differ from each other in efficiency and the required duration, as well as in the properties and differentiating potential of the resulting NSCs (Watanabe et al., 2007; Chambers et al., 2009; Kim et al., 2011; Fedorova et al., 2019). Approaches using neural differentiation of PSCs in 2D cultures have several limitations. For example, in plastic dishes without physiological adhesion substrate, the cells of the 2D culture interact more with the plastic than with each other, even when in neural rosettes. In addition, the distribution and gradients of morphogens and signaling molecules that the cells exchange during differentiation are dispersed and are very different from those *in vivo*.

Three-dimensional cultures – the highest level of complexity – include the use of neural spheroids and organelles. This approach is the best for modeling neurogenesis because it more closely follows the spatiotemporal, *in vivo* development of the nervous system. Three-dimensional neural cultures can reproduce the intercellular interactions, cytoarchitecture, and the complete variety of cell types, and, therefore, more accurately reflect the morphogen gradients. Such neural cultures can be maintained for extended periods (up to 2 years) (Eiraku et al., 2011; Lancaster et al., 2013). However, this approach usually requires special equipment, such as spin bioreactors (Qian et al., 2016). The study of some processes can also be difficult due to the high level of heterogeneity. In addition, 3D methods are not suitable for the direct generation of NSCs due to difficulties in isolating the cells. By contrast, monolayer culture and the neural rosette approaches (without an EB step) are suitable for routine NSC generation for imaging assays, genomic screening and morphological studies. They can also supply pure NSC cultures for therapeutic transplantation studies. 3D culture, instead, provides a platform for the study of cell–cell interactions, the diversity of neural cells, and for investigations of *in vivo* neurogenesis and the development of neurological diseases (Lancaster et al., 2013; Kim et al., 2015*;* Gonzalez et al., 2018; Chlebanowska et al., 2020). Both 2D and 3D cultures are useful for drug testing, but, while 3D culture allows for useful study of the overall complex effects of substances, 2D culture provides greater convenience for more detailed study of individual processes.

Induced pluripotent stem cell-derived NSCs and their differentiated derivates can have applications in various fields from the investigation and modeling of neurological diseases and drug screening to the treatment of neurodegenerative diseases. Transplantation of NSCs or differentiated neurons into patients with neurodegenerative diseases seems to be a very promising approach in disease treatment. However, there are many questions and concerns about the efficiency and safety of such procedures. For example, for transplantation, the cell population must be pure and have well-defined characteristics. To achieve this, the protocols for obtaining NSCs from iPSCs should be strictly standardized and easily reproducible. Furthermore, before clinical trials, the treatment approach should be tested on animal models. Unfortunately, the animal models of human neurological pathologies have significant limitations (Adami et al., 2014).

Because of difficulties of the animal modeling of human neurological diseases, *in vitro* models have an advantage and can be used for pathology investigation and for testing drug treatment approaches. Over the past few years, *in vitro* models of diseases such as Alzheimer’s disease (AD), Parkinson’s disease, Huntington’s disease, spinal muscular atrophy and others, have been actively developed. The most popular models are those based on the differentiation of patient-derived iPSCs into NSCs and their differentiated derivates (Israel et al., 2012; Mehta et al., 2018) or into brain organoids (Gonzalez et al., 2018; Virlogeux et al., 2018; Chlebanowska et al., 2020). It should be noted that the different protocols of NSC generation from hiPSCs may result in slightly different characteristics of the created models of certain diseases. Nevertheless, in general, the different approaches to modeling the same disease can provide phenotypes of interest. As useful example, we can compare three different protocols for obtaining NSCs and neurons from iPSCs from patients with familial AD caused by the A246E mutation in the PSEN1 gene. For NSC generation, Mahairaki et al. (2014) applied the Dual SMAD inhibition protocol to EBs formed from patient-derived iPSCs, and the differentiation of neurons was initiated by the action of the factors BDNF, GDNF, and cAMP. Armijo et al. (2017) used the NSC differentiation protocol under 2D conditions developed by Shi et al., using CHIR99021, LIF, SB and Compound E. (Shi et al., 2012), followed by neuronal differentiation initiated by BDNF, GDNF, and cAMP (Armijo et al., 2017). Gonzalez et al. (2018) used a model of AD based on cerebral organoids from patients’ iPSCs, induced with the DUAL SMAD inhibition protocol. In cultures from all three models, neurons expressing MAP2 and βIII-tubulin were detected, while having specific AD-like phenotypes. The secretion of amyloidogenic variants of the amyloid-beta (Aβ) (1–42 and 1–40) peptides were increased, as was their Aβ(1–42)/Aβ(1–40) ratio; increased tau phosphorylation was also observed. These models differed in cultivation duration. Differentiating cerebral organoids takes significantly longer, however, such 3D models have advantages. The fact is, that iPSC-based models provide neurons with an immature, fetal phenotype, yet AD usually represents an age-dependent progressive disease. Therefore, 3D organoids, by providing the development of more mature neurons, brings this *in vitro* model closer to reality. Since cerebral organoids can be maintained in culture for a very long time, the accumulation of Aβ aggregates resembling plaques can be observed in such a model (Lancaster et al., 2013; Gonzalez et al., 2018). Depending on the goals, various modeling options for neurodegenerative diseases based on iPSCs can be used, and each model will have its own advantages.

Tables 1, 2 provide summaries of the main protocols of NSC induction from PSCs.

## Conclusion

The development of NSC generation protocols has been continuing for a substantial time. To date, there are many different protocols that differ in the conditions and duration required for cultivation. On the one hand, the diversity of protocols for obtaining NSCs from PSCs *in vitro* provides a better understanding of the conditions needed for neural induction, and a detailed study of the mechanisms, but on the other hand, this has led to the generation of a huge variety of NSC phenotypes. Different NSC lines exhibit different proliferative capacity and differentiation potentials. Even small deviations in the culture conditions, concentrations of exogenic factors, and their time of exposure can affect the differentiation of the PSCs, the phenotypes of the NSCs, and the fate specification. And, while this may not be so critical for the study of neurogenesis, it *is* important in the modeling of diseases, and even more so for cell therapy, where the standardization of NSC lines and their reproducibility is necessary. For therapeutic applications, NSCs must be homogeneous, stable self-renewable cultures with defined characteristics of the multipotent stem cells. That is why it is necessary not only to develop one or several universal and reproducible protocols for obtaining NSCs *in vitro* from PSCs but also to select universal criteria for evaluating the NSC phenotypes and the methods for their selection.

## Author Contributions

AG researched and wrote the manuscript and prepared the figure and tables. ED read, edited, and approved the manuscript. Both authors contributed to the article and approved the submitted version.

## Conflict of Interest

The authors declare that the research was conducted in the absence of any commercial or financial relationships that could be construed as a potential conflict of interest.
